# Providers and Patients Caught Between Standardization and Individualization: Individualized Standardization as a Solution: Comment on "(Re) Making the Procrustean Bed? Standardization and Customization as Competing Logics in Healthcare"

**DOI:** 10.15171/ijhpm.2017.95

**Published:** 2017-08-12

**Authors:** Lena Ansmann, Holger Pfaff

**Affiliations:** Institute of Medical Sociology, Health Services Research and Rehabilitation Science, Faculty of Human Sciences and Faculty of Medicine, University of Cologne, Cologne, Germany

**Keywords:** Standardization, Customization, Individualization, Guidelines, Decision-Making, Tumor Conferences

## Abstract

In their 2017 article, Mannion and Exworthy provide a thoughtful and theory-based analysis of two parallel trends in modern healthcare systems and their competing and conflicting logics: standardization and customization. This commentary further discusses the challenge of treatment decision-making in times of evidence-based medicine (EBM), shared decision-making and personalized medicine. From the perspective of systems theory, we propose the concept of individualized standardization as a solution to the problem. According to this concept, standardization is conceptualized as a guiding framework leaving room for individualization in the patient physician interaction. The theoretical background is the concept of context management according to systems theory. Moreover, the comment suggests multidisciplinary teams as a possible solution for the integration of standardization and individualization, using the example of multidisciplinary tumor conferences and highlighting its limitations. The comment also supports the authors’ statement of the patient as co-producer and introduces the idea that the competing logics of standardization and individualization are a matter of perspective on macro, meso and micro levels.

Mannion and Exworthy^1^ provide a timely discussion on the seemingly contradictory trends towards both the standardization and the customization of healthcare and medical treatment. With this paper, the authors make an important contribution to an understudied topic of health services research. The authors describe and define standardization and customization within healthcare and discuss the implications of both parallel trends for healthcare delivery based on sociological theory. Specifically, in analogy to the Greek myth of the Procrustean bed, arbitrary standardization is described as forced conformity.

## Customization, Personalization and Individualization – What Is the Difference?

In contrast to Mannion and Exworthy, we suggest to differentiate the terms customization, personalization and individualization in healthcare (see Figure). Whereas customization is the tailoring of standardized treatment and diagnosis to the psychological, social, and cultural dimensions of the patients, eg, patient preferences and wishes, personalization in medicine means the adaption of treatment to the biological dimensions of the patient’s body. Thus, personalization means that “medical care can be tailored to the genomic and molecular profile of the individual.”^2^ The term individualization can be used as the umbrella term for the adaption of health services to the patient’s biological, psychological, social and cultural dimensions.^3^

**Figure:**
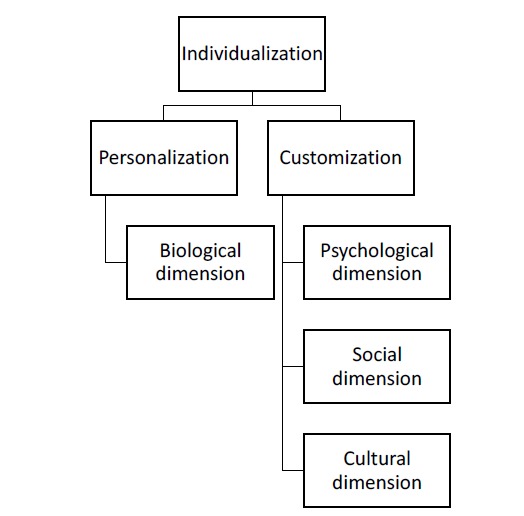
Conceptual Differentiation Between Individualization, Personalization, and Customization in Healthcare.

## Drivers of Individualization

Around 25 years ago, the idea of evidence-based medicine (EBM) was spread. Whereas at first providers were skeptical of the concept of grounding treatment decisions largely on research evidence instead of clinical experience, the paradigm of EBM is now widely accepted. EBM facilitated standardization in healthcare, eg, by developing and implementing clinical practice guidelines. Nowadays, in times of personalized medicine, a “one fits all” type of treatment has its limits.^4^ Patient care is increasingly customized or personalized as a result of three trends. Firstly, the growing scientific knowledge facilitates precision medicine due to highly specific individual diagnostics and tailored therapies.^4,5^ Secondly, the increasing number of multimorbid patients urges deviation from evidence-based guidelines, since guidelines seldom address the co-occurence of two or more diseases.^6^ Thirdly, patient empowerment urges shared decision-making between provider and patient.^7^ As a consequence, the use of research evidence in treatment decision-making is changing: more individualization of medical treatment to individual patients is required. In healthcare practice, providers and patients face the task to integrate up-to-date knowledge and patient preferences in decisions concerning individual patients, while under many pressures and constraints from the healthcare system.^7^

Clinical practice guidelines are the prime example for a widespread tool for standardization in healthcare. The authors address some of the issues regarding the use and usefulness of these guidelines in practice,^8^ and thereby, describe the conflict between standardization and individualization. Besides the sometimes-insufficient and quickly changing evidence base of guidelines and problems with acceptance and adherence among providers,^9-13^ it is important to mention the limitations of evidence-based guidelines per se. Guidelines cannot encompass evidence-based recommendations for every potentially emerging case. Guidelines can only inform recommendations for the most common types of cases. For specific and complex cases, including multimorbid patients, the experience and tacit knowledge^14^ of the provider (the authors call it “mindlines”), as well as the patient’s conditions and preferences, must play an even bigger role in decision-making.^7^ Whereas standards provide the rules of thumb, individualization provides a construct to fill-in the gaps in knowledge for specific cases. Thus, standardization has its natural limitations, and deviations from guideline recommendations for specific cases may be necessary and beneficial, as shown in a recent study from Germany.^15^ In these cases, formal sanctioning deviating provider behavior will be harmful to providers and patients.^3^

Mannion and Exworthy outline a well-justified need for research on the strategies of deciding between, or balancing, standardization and individualization in everyday healthcare. Nowadays, one of the provider’s main tasks is to break down standards to the individual patient and not to force conformity. Like the authors stated, medicine is always individual opposed to public health focusing on populations. But evidence from clinical studies usually is not reported for every single subgroup of patients. Within the consultation, the provider needs to transfer evidence-based knowledge on populations to the individual patient.^7^ Thus, in the following we propose a concept of individualized standardization as a solution to the described challenge.

## The Concept of Individualized Standardization

Individualized standardization of care “is defined as the imposition of standards, regulations or norms which are tailored to the genes, body condition, culture, social environment, values, needs and preferences of the individual patient.”^3^ Individualized standardization is conceptualized as a guiding framework leaving room for individualization in the patient-physician interaction system, which can be regarded as an autopoetic target group. The term “autopoiesis” was shaped by Niklas Luhmann, a German sociologist and co-founder of systems theory^16^ and describes the process of self-preservation of social systems. A system is autopoetic, when it constantly and non-purposefully reproduces itself from within itself. Individualization, as described above, means taking into account the biological, psychological, social and cultural dimensions of the patient, eg, patient preferences and wishes. The theoretical background is the concept of context management according to systems theory. “Context management can be viewed as a technique that creates an environment in which an autopoetic target group is more sensitive to steering signals. Context management is a technique making use of the self-referential closure of the autopoetic target group.”^17^ Transferred to healthcare, context management creates an environment in which the patient-physician interaction is sensitive to steering signals, eg, guideline knowledge, quality management activities, expertise from other disciplines and molecular diagnostics. Within this context treatment has to be individualized within a given framework (ie, standardization). The aim is to merge evidence with patient preferences. As Barratt argues,^7^ this ideal aim could only be reached by intensive training. For her it is “really important (…) that future doctors are trained to individualize treatment to patients – because that is necessary for doing a good job of both EBM and SDM [shared decision making].” And she adds: “we should not have a view that good practice requires doctors – and patients – to follow or comply with guidelines.”^7^ One example for the realization of the idea of merging individualization and standardization in clinical practice are multidisciplinary tumor conferences (MTCs). MTCs can be regarded as autopoetic groups with the tendency to self-referential closure.

## Individualized Standardization in Multidisciplinary Tumor Conferences

One example for the integration of standardization and might be multidisciplinary teams in oncology. MTCs have been established to deal with the complexity of oncological care and to support treatment decision-making. MTCs are regular meetings of a multidisciplinary treatment team in which the diagnosis, prognosis and treatment of cancer patients are discussed.^18^ In MTCs, treatment recommendations are not only to be made based on evidence and standardized protocols, but also on input and perspectives from multiple disciplines on the individual patient. Here, deviations from guidelines are possible, but need to be reasonably explained. These deviations are in most cases results of the individualization of standardized procedures and can be beneficial for the individual patient.^15^ The patients’ conditions (eg, comorbidities, social environment) and their wishes and preferences should be considered and inform the recommendation, as well. Admittedly, the composition of disciplines participating in MTC is selective, too. Nurses, who commonly have the best knowledge about the patients’ social environment and preferences and thus could act as a patient’s advocate, seldom take an active part in the MTC.^19^ For this reason, some clinic began to invite patients to take part in their own MTC.^18^ On the one side, studies reveal benefits of MTCs for treatment decision-making,^20^ and MTCs are accepted and valued by providers and patients.^21,22^ On the other side, studies show that the patients’ characteristics and perspectives are seldom considered in MTCs^19,23,24^ and recommendations from the MTCs are not always followed by patients and providers.^23^ In summary, the idea of MTCs is somewhat representative of the concept of individualized standardization.^3^

## The Patient as Co-producer

Another natural limitation to standardization in healthcare are the patients, themselves. In human services, the client - here the patient - needs to cooperate in order to fulfill the service. As mentioned by Mannion and Exworthy, the patient is a “co-producer.” In healthcare, this is especially relevant, since treatment success is strongly dependent on the patient’s cooperation, adherence and trust.^25-27^ Moreover, patients need to give their explicit consent to medical procedures. Thus, even if the evidence-based standard was recommended to the patient, the patient can choose a different treatment, can refuse treatment, and does not eg, have to take the prescribed medication. And patients can get a second opinion from a different provider before deciding, which again ensures quality and standards. Furthermore, financial constraints of the patients and reimbursement policies for providers may play a central role in treatment decision-making, too.^28^ Hence, the patients’ behavior in decision-making cannot be completely controlled by standardization.^29,30^

## Personalized Medicine as a Challenge to Shared Decision Making and the Healthcare System

The authors describe the trend towards individualization by using the example of personalized medicine, which we will be confronted with in the future even more, as described above. Personalized medicine uses big data, eg, oncological databases such as CancerLinQ, Flatiron and IBM Watson, in order to identify differentiated patient profiles, which are used to decide on highly-targeted and effective therapies.^31^ Big data can help to identify promising treatment options based on the patient’s biology, but still the patient needs to be empowered to understand the options and participate in the treatment decision-making. Due to the complexity of the options, it will be even more challenging for providers to inform and involve patients in treatment decisions. From a systems’ perspective, a central question arises: How can we regulate personalized medicine in our healthcare systems and how can the costs be handled? The need for regulation stems from the need to ensure high quality care and prevent harm, as well as from the need to restrict utilization of services in personalized medicine due to financial constraints of the healthcare and insurance systems. In this regard, research and policy are urgently required to develop feasible and fair solutions. First studies reveal that personalized medicine is cost-effective due to limiting the use of eg, targeted drugs to patients with a certain well-defined risk profile, for which the drugs are highly effective.^31^

All in all, we suggest that the balance between standardization and individualization is a matter of perspective. As the authors already point out, both trends play out on the macro, meso and micro levels. The macro level’s interests (society and healthcare system) in healthcare are concerning cost-effectiveness, reducing variation between providers, affordability and quality assurance; thus, the system potentially favors standardization as a form of regulation and context management. On the meso level (healthcare organizations), the interests are mostly on high-quality care, competition, cost-effectiveness and the use and accumulation of resources. Hence, standardization and individualization should best be balanced to reach these interests. On a micro level, providers and teams may focus on treatment success, patient well-being, ethical care, competition and autonomy; whereas patients’ interests may depend on, for example, their needs and preferences, treatment success, side effects and trust. On the micro level individualization may be more important than standardization, whereas on the macro level standardization is key. All in all, the question that still needs to be answered on all levels is: How do stakeholders in healthcare balance or decide between standardization and individualization together with their patients?

## Ethical issues

Not applicable.

## Competing interests

Authors declare that they have no competing interests.

## Authors’ contributions

LA and HP both contributed to the conception of the article, the drafting and the critical revisiting of the article.
